# Sentiment Diffusion of Public Opinions about Hot Events: Based on Complex Network

**DOI:** 10.1371/journal.pone.0140027

**Published:** 2015-10-13

**Authors:** Xiaoqing Hao, Haizhong An, Lijia Zhang, Huajiao Li, Guannan Wei

**Affiliations:** 1 School of Humanities and Economic Management, China University of Geosciences, Beijing 100083, China; 2 Key Laboratory of Carrying Capacity Assessment for Resource and Environment, Ministry of Land and Resources, Beijing 100083, China; 3 Lab of Resources and Environmental Management, China University of Geosciences, Beijing 100083, China; Universitat Rovira i Virgili, SPAIN

## Abstract

To study the sentiment diffusion of online public opinions about hot events, we collected people’s posts through web data mining techniques. We calculated the sentiment value of each post based on a sentiment dictionary. Next, we divided those posts into five different orientations of sentiments: strongly positive (*P*), weakly positive (*p*), neutral (*o*), weakly negative (*n*), and strongly negative (*N*). These sentiments are combined into modes through coarse graining. We constructed sentiment mode complex network of online public opinions (SMCOP) with modes as nodes and the conversion relation in chronological order between different types of modes as edges. We calculated the strength, k-plex clique, clustering coefficient and betweenness centrality of the SMCOP. The results show that the strength distribution obeys power law. Most posts’ sentiments are weakly positive and neutral, whereas few are strongly negative. There are weakly positive subgroups and neutral subgroups with *ppppp* and *ooooo* as the core mode, respectively. Few modes have larger betweenness centrality values and most modes convert to each other with these higher betweenness centrality modes as mediums. Therefore, the relevant person or institutes can take measures to lead people’s sentiments regarding online hot events according to the sentiment diffusion mechanism.

## Introduction

With the development of Internet, millions of web users spend hours a day on the website, especially on social networks. By December 2013, the number of Chinese internet users reached 618 million. The usage rate of social web is 45.0% (CNNIC33). People read news and write their views and opinions about online hot events and commodities [1]. Therefore, people’s posts constitute online public opinions. These opinions and views reflect people’s sentiments. The sentiments in social networks can affect people’s purchase behavior [2], the sellers’ marketing plan [3], political trends [4, 5] and effectively forecast stock market [6]. Moreover, many online hot events change trends with time going by. With the development of the events and increasing comments, the sentiment of users contained in the comments influences each other. Thus, the sentiment of public opinions diffuses through the internet. Meanwhile, the relevant persons or institutes may change decisions to deal with the event according to the sentiment diffusion. Thus, the sentiments of online public opinions may have some laws with relation to time and mutual infection. The exiting work on social network sentiment just to distinguish the sentiment polarity of public opinion [7]. However, they didn’t consider the sentiment diffusion mechanism. In fact, the former post will affect the sentiment of the latter post. Thus, there is sentiment diffusion mechanism in the public opinion. Therefore, we are going to study the diffusion mechanism of different sentiment orientation implicated within online public opinion. Thus, the relevant person or institute can take measures to prevent potential crises caused by violent sentiments in time.

In order to get different sentiment orientation of posts, we need to apply the sentiment analysis approach, whose one of purposes is to classify the attitude expressed in the text (such as positive or negative) [8]. Currently, most sentiment analysis techniques can be divided into machine-learning approaches and dictionary-based approaches. Machine-learning approaches based on using a collection of data to train the classifiers [9]. The compilation of these training data requires considerable time and effort, especially since data should be current [10]. Meanwhile, dictionary-based approaches extract the polarity of each sentence in a document, which have important advantages, such as the fact that once they are built, no training data are necessary. The most frequently used resources are currently SentiWordNet and WordNet which have been employed in a large number of research studies [11–13]. Therefore, to obtain the sentiment orientation of Chinese, we adopted dictionary-based approach based on HowNet.

The sentiment analysis approach mentioned above provides basis for our research. As for sentiment diffusion, the former sentiment can affect the latter one, from one type sentiment to another one will pass media and there will be some sentiment emerging in a focus time. Hence, the sentiment diffusion of public opinion is a dynamic complex process. Complex network theory provides a theoretical approach to study the complexity science. The core of complex network is to reveal the feature of complex system by its structure. Complex network theory has been applied to business economics [14, 15], social sciences [16], international trade [17], and text mining [18]. And the complex network could influence some special system [19]. In terms of using a complex network to research online public opinion, Kwak et.al [20] and others discussed the structure and characteristics of micro-blog social networks by analyzing the Twitter network and comparing micro-blog to traditional online communities. Krishna et.al [21] analyzed the features of Twitter users in terms of fans and attention numbers. Huberman et.al [22], using Twitter as an example, analyzed the centrality of micro-blog social networks by observing a group of micro-blog users. Therefore, we applied complex theory to identify the main sentiment status, the main sentiment dissemination pattern and media to reveal the sentiment diffusion mechanism about online public opinion.

The paper is organized as follows: After the introduction in the first section, the second section introduces the methodology and data used in this paper. The third section discusses the results and analysis, which include the sentiment distribution and dissemination of online public opinions. Section four presents the conclusion and future work.

## Methodology and Data

The method is separated into three parts. First of all, we converted the abstract sentiment of posts into specific sentiment symbol series. Secondly, we separated the continuous sentiment symbol series into sentiment fragments as well as sentiment modes by coarse graining. The sentiment modes represent different kinds of sentiment diffusion status. Last, we constructed sentiment modes network and analyzed its structure.

### Sentiment symbol of online public opinions

There are many studies on sentiment analysis techniques for online public opinion [23–25]. Scholars set weights to the sentiments of text to precisely quantitatively describe the sentiments that people have [26, 27]. We quantify the sentiments of people’s posts using sentiment analysis technique. These sentiments are divided into five kinds of sentiment, i.e., strongly positive, weakly positive, neutral, weakly negative and strongly negative for analyzing the sentiment of online public opinions. We propose a sentiments symbol collection approach which has three steps. Step 1aims to construct a sentiment dictionary with sentiment value. In step 2, we calculated the sentiment value of each post. In step 3, we divided different sentiment orientation of posts to obtain the sentiment symbols. More descriptions of this approach are as follows:

#### Step 1: Constructing the sentiment dictionary

Determining the sentiment values of people’s posts needs lexical semantic analysis technology. According to a positive and negative sentiment vocabulary dictionary and the computing capability of semantic similarity provided by HowNet [28], we build a sentiment vocabulary dictionary that each word has a sentiment value. In the HowNet, there are 3116 negative words and 3730 positive words. The sentiment orientation of these words is fixed and clear.

For any word, we can obtain its sentiment value by calculating its semantic similarity with two basic groups of words whose sentiment orientations are very clear, strong and representative words. In the two groups, the seed words are selected artificially and each word has a clear orientation: one group expresses positive sentiments, and the other expresses negative sentiments. The sentiment value of positive sentiment words is calculated following Eq 1 while the sentiment value of negative sentiment words is calculated following Eq 2.

$$S_{W1}=\frac{1}{n}\sum_{i=1}^{n}sip(word,seed_{1i})$$ (1)

$$S_{W2}=-\frac{1}{p}\sum_{i=1}^{p}sip(word,seed_{2i})$$ (2)

Where *seed*_*1*_ represents the positive-seed words and *seed*_*2*_ represents the negative-seed words. The numbers of group words are *n* and *p*, and the function *sip (word*, *seed)* calculates the semantic similarity between two words.

In this article, semantic similarity is based on the machine translation. Semantic similarity refers to the degree which the two words in different contexts can replace each other without changing the syntactic semantic structure. Two words, if the chance that in different contexts they can replace each other and do not change the syntactic semantic structure is larger, their similarity is higher, and vice versa [29].

HowNet is a common sense knowledge base, in which objects are the concept represented by Chinese and English words and basic content is to reveal the relationship between concepts and nature of concepts. For Chinese vocabulary, the description of words base on the "primitive" in HowNet. Primitive can be considered to be the most basic and not ease to separate smallest semantic unit in Chinese. Because the meaning of "word" in Chinese is very complex, a word in different context often will express different semantics [30]. Therefore, in HowNet, Chinese word is a collection of several primitives. For two Chinese words *W*_*1*_ and *W*_*2*_, if *W*_*1*_ have *n* primitives (concept): *S*_*11*_, *S*_*12*_,*……*, *S*_*1n*_, *W*_*2*_ have *m* primitives (concept): *S*_*21*_, *S*_*22*_, *……*, *S*_*2m*_. The similarity of *W*_*1*_ and *W*_*2*_ is the maximum similarity of each primitive similarity using Eq 3. [31]

$$Sip(W_{1},W_{2})=\max_{i=1\ldots n,j=i\ldots m}sip(s_{1i},s_{2j})$$ (3)

In HowNet, there are eight kinds of relationship between primitives: up and down relationship, synonymy relationship, antonym relationship, righteousness relationship, attribute-host relationships, part-overall relationship, material-finished products relationship, and events-role relationship. Thus, relationship between primitive is a complex network structure, rather than a simple tree structure. However, the up and down relationship is still the most important one. According to the up and down relationship, all the basic primitives form a hierarchy system as a tree structure.

The similarity of meanings is their semantic distance. Assuming two meanings path distance in the HowNet hierarchy system is *d*. Their similarity is calculated as Eq 4. [31]

$$Sip(p_{1},p_{2})=\frac{\alpha }{d+\alpha }$$ (4)

where *p*_*1*_ and *p*_*2*_ are two primitives, *α* is an adjustable parameter which means that the distance between words when the similarity is 0.5.

The experimental results show that the larger number the seed words have, the higher the accuracy of the judgment will be. In this paper, the number of positive seed words, *n*, is 60, and the number of negative seed words, *p*, is 60. As above said, we selected strong and representative sentiment orientation words. These 120 seed words fit the requirement. In the sentiment word polarity identification experiment, it obtained the good experimental results which can reach 99 percent [32].

Degree adverbs will strengthen or weaken the strength of the sentiment of words. When we calculate the sentiment value, we should not only consider the sentiment of the word itself but also the role of the degree adverbs. We give a different degree value to each degree adverb. The negative adverb changes the orientation of sentiment to its opposite side. Thus, the degree value of negative adverb is -1. Some of the degree values from the degree adverbs dictionary are shown in Table 1.

**Table 1:** Some words’ degree values in the degree adverbs dictionary.

words(Chinese phonetic alphabet)	degree values	words(Chinese phonetic alphabet)	degree values	words(Chinese phonetic alphabet)	degree values
Very (hen)	1.4	Totally (wan quan)	1.9	Slightly (lue wei)	0.6
More (geng)	1.7	Double (bei jia)	1.8	a little (shao)	0.6
Extremely (ji qi)	1.9	the more (yue fa)	1.5	a little (shao wei)	0.6
Utterly (shi fen)	1.8	Fully (chong fen)	1.8	a little (shao xu)	0.6
Reasonably (xiang dang)	1.7	Excessively (guo yu)	1.4	Pretty (ting)	0.5
extremely (ji du)	1.9	Highly (fei chang)	1.8	no/not (bie)	-1
Utmostly (ji)	1.9	Extremely (ji duan)	1.9	no/not (wu)	-1
no/not (bu)	-1	no/not (mei)	-1		

#### Step 2: Calculating the sentiment value of each post

When we collect standardized information about posts, *Sen*_*p*_ is each post, and $O_{Sen_{p}}$ is the sentiment value. To calculate the sentiment value of each post, the post needs to be separated into words, because there is no space between Chinese words. Therefore, we need segmentation techniques in the natural language. We also need to know the part of speech of every word because most of the sentiment words are adjectives. We have to extract the adjective adverbs near adjectives, which are used to determine the degree of the adjectives. We used open source software "Jieba" (Chinese for "to stutter") to segment Chinese text, which is built to be the best Chinese word segmentation module in Python. The segmentation algorithm is based on Tire tree and maximum probability path, for unknown words, the character position HMM-based model and Viterbi algorithm is used. The license of Jieba is MIT License. We extracted sentiment words in every post, which had been separated. If one or two adverbs appear in front of the sentiment word, i.e., *Wi*, with *Advi1* and *Advi2* being degree adverbs in the dictionary, the final sentiment value of each sentiment word is shown in Eq 5. [33]

$$o_{wi}=p_{advi_{1}}\;*\;p_{advi_{2}}\;*\;s_{wi},$$ (5)

Where $p_{advi_{1}}$ represents the sentiment value of degree adverbs and *s*_*wi*_ represents the sentiment value of *Wi*. For each post *Sen*_*p*_, if it has *k* sentiment words and each of them is denoted as symbol *w*_1_, *w*_2_,⋯, *w*_*k*_, the final sentiment value of each post *Sen*_*p*_ is $O_{Sen_{p}}$, as shown in Eq 6. [33]:

$$o_{sen_{p}}=\frac{1}{k}\sum_{i=1}^{k}o_{wi}$$ (6)

#### Step 3: Classifying the sentiment category

We calculated the cumulative sentiment value of posts $O_{Sen_{p}}$ and get the cumulative distribution of posts’ absolute sentiment value. About 97% posts’ absolute sentiment value is below between 0 and 1. About 54% posts’ absolute sentiment value is 0, and 10% is bigger than 0.8. The strongly positive or negative sentiments are strong emotions. Therefore, we select ‘+/-0.8’ as the threshold. We have hand label couple of hundreds posts and test the accuracy of the sentiment lexicon constructed. We listed some cases in Table 2. From the cases we can see that the sentiment analysis method can get relative good result.

**Table 2:** Sentiment Analysis Evaluation Cases.

Posts	O_senp_	Sentiment Value by Hand
Strongly support the building-owner	1.5	1.7
The actions of TX makes netizens feel sick, it is a wolf in sheep's clothing	-0.8	-1.2
I support too.	0.8	0.8
Strongly support the building-owner. I don’t have money, but I have spirit to support.	1.5	1.6
I agree.	0.8	0.8
Tencent! Damn it!	-0.7	-0.8
Totally support.	1.4	1.5
Strongly condemn Huateng Ma.	-1.2	-1.4
Count me in! Strongly support 360!	1.4	1.6
Must be someone stand up! Strongly despise Tencent! Free competition! Give the option to the user!	-1.2	-1.4

Based on the value of $O_{Sen_{p}}$, we define the sentiment degree of internet users’ posts and abstracted the sentiment degree to symbols *C*_*i*_. The division standard of the sentiment degree can vary according to the research requirements. We define the sentiment categories as in Eq 7.

$$c_{i}=\left\{\begin{array}{l} P\;(o_{sen_{p}}>0.8,\;\text{strongly positive})\\ p\;(0<o_{sen_{p}}\leq 0.8,\;\text{weakly positive})\\ o\;(o_{sen_{p}}=0,\;\text{neutral})\\ n\;(\text{-}0.8\leq o_{sen_{p}}<0,\;\text{weakly negative})\\ N\;(o_{sen_{p}}<0.8,\;\text{strongly negative}) \end{array}\right\}$$ (7)

We rank the sentiment symbols *C*_*i*_ of posts into corresponding sentiment series in chronological order, as shown in Eq 8.

$$C_{T}=(c_{1}c_{2}c_{3}\cdots )\;({\text{c}}_{\text{i}}\in (\text{P},\text{p},\text{o},\text{n},\text{N}))$$ (8)

### Sentiment modes by coarse graining

To construct complex network based on the sentiment symbol series, we applied coarse graining. Hence, the sentiment symbol series is transferred into a series of sentiment modes. We adopted "sliding window" to get the sentiment modes. Some studies like coarse potentials shift among temperatures [34], dynamics simulation analysis [35], economic time series [36] and molecular electrostatic coupling [37], using coarse graining to study complex systems. Coarse graining processing makes homogeneous divisions among intervals [38]. The entire system interval is divided averagely into finite sub-intervals given a string. In the conversion process, there is information transfer or control function between the modes, and each mode is based on several modes before it, such that the sentiment mode of online public opinions has memory and transmission as well as pluralist features [39]. Therefore, the contact pattern between modes is direct, and the extent of contact varies. As a result, different types of contact constitute the sentiment complex system of online public opinions.

It is worth noting that the validity of the results is largely dependent on the accuracy of coarse graining. To better analyze the sentiment changes of online public opinions, the number of combinations of coarse graining symbols should not be too large. For this reason, we selected four, five and six symbols as one unit and, in step 1 for "data sliding window", combined every four, five and six sentiment symbols, respectively, into one sentiment mode. It turns out that each five symbols as a unit can better reflects the sentiment diffusion. The coarse graining process is shown in Table 3. For instance, the sentiment mode "*PPPPP*" means that the sentiment status is continuous strongly positive. The sentiment mode "*NNNNN*" means that the sentiment status is continuous strongly negative.

**Table 3:** Coarse graining processing of sentiment modes.

serial number	sentiment series	Coarse graining mode
1	*P*	
2	*P*	
3	*P*	
4	*p*	
5	*p*	*PPPpp*
6	*o*	*PPppo*
7	*o*	*Pppoo*
8	*N*	*ppooN*
……	*……*	……

### The complex network of sentiment modes

**Fig 1:**
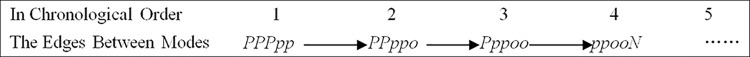
Constructing edges between modes in SMCOP. The arrow represents the edges between modes.

We construct an online public opinion sentiment mode weighted directed complex network (SMCOP) with modes as nodes, the conversion relation in chronological order between different types of modes as edges. The direction of edges is from the former one to the latter one. In the network, if there is edge between two modes, it means that the two modes appear in time order. If mode *i* appears before mode *j*, then a link from *i* to *j* is drawn and a_*ij*_ = 1. The conversion frequency from mode *i* to mode *j* is the weight of their edge denoted as *w*_*ij*_. (Fig 1). Thus, the study of the sentiment diffusion of online public opinions can be transformed into study the structure characteristics of the SMCOP.

Complex network analysis theory provides many parameters, including node strength, the weighted clustering coefficient, average shortest path and betweenness centrality. We will analyze the sentiment mode diffusion of online public opinions, which includes its distribution and dissemination, through those parameters.

#### (1) The sentiment mode distribution of online public opinions

We identified the main sentiment modes in the sentiment diffusion process by node strength. We explored the overall sentiment diffusion situation by node strength distribution.

Node strength not only considers the number of neighbors of a node in the network but also the weight between each node and its neighbor nodes. Therefore, the node strength is the comprehensive embodiment of the local information of nodes [40]. For the directed network, assume the number of nodes connected to node *i* is *n* and the weight of node *i* to node *j* is *w*_*ij*_, the node strength *s*_*i*_ of node *i* is defined by Eq 9:

$${\text{s}}_{\text{i}}=\sum_{j=1}^{n}\left(w_{ij}+w_{ji}\right).$$ (9)

The node strength distribution *P (s)* is defined by Eq 10.

$$p(s)=\frac{s_{i}}{N}$$ (10)

Where *N* is the sum of all nodes’ strength and *s*_*i*_ is the node strength.

Eq 9 shows that the greater the node strength is, the more frequent the conversion between this node and other modes will be. As a result, this fluctuation mode is more important in the SMCOP.

#### (2) The sentiment mode dissemination of online public opinions

To be more specific, we analyzed the main sentiment dissemination pattern, core and medium through k-plex, weighted clustering coefficient and betweenness centrality.

Frequently mutual conversion between modes will form a small relation network, i.e., network subgroups. The discovery of network subgroups could use k-plex method which is employed to discover the subgroups based on the node degree. The principle of the k-plex method is as follows: Each point in the sub-group is directly connected with other nodes in addition to *K* nodes (i.e., if a cohesion subgroup’s scale is *n*, then only when the degree of each node in the sub-group is less than the value of (*n-k*), the sub-group is a k-plex). When the value of *k* is smaller and the values of *n* are greater, the conditions are harsher and there are more conversion patterns between modes [41]. By discovering subgroups, this paper can answer the questions of which modes make mutual conversions and what is the conversion pattern.

However, for exploring the core mode in transformation processing also requires analyzing the clustering coefficient of the network. The clustering coefficient of a node reflects the correlative degree between neighbors of the node. Because the SMCOP is a weighted network, we use the weighted clustering coefficient $c_{i}^{w}$ to calculate it. The weighted cluster coefficient $c_{i}^{w}$ is defined by Eq 11 [42].

$$c_{i}^{w}=\frac{1}{k_{i}\;*\;(k_{i}-1)}\sum_{j,k}{\left(w_{ij}^{n}w_{jk}^{n}w_{ki}^{n}\right)}^{\frac{1}{3}}$$ (11)

Where *k*_*i*_ is the degree of node *i*. And *w*_*ij*_ *w*_*jk*_ and *w*_*ki*_ are the weight of edges between two nodes (*i*, *j*), (*j*, *k*) and (*k*, *i*). Eq 9 shows that the larger the weighted clustering coefficient of one node is, the more closely the conversion among neighbors of this mode will be. As a result, this mode is more important in the subgroup.

The conversion among different modes and subgroups need to go through some media. We use betweenness centrality to analyze which modes are in the intermediate position in the SMCOP. Assume that there are *n* shortcuts between node *X* and node *Z*. Specific to the SMCOP, a mode *Y*’s betweenness centrality relative to mode *X* and mode *Z* refers to the ability of this mode in the shortcut of these two modes. Betweenness centrality measures the control of sentiment mode dissemination of one mode. The greater the betweenness centrality the one mode has, the more important the mode in sentiment dissemination will be. The betweenness centrality of node *i* is calculated by Eq 12:

$$c_{RBi}=\frac{2c_{ABi}}{c_{Pax}}$$ (12)

where *c*_*ABi*_ represents the absolute betweenness centrality of the node, which is denoted as $c_{ABi}=\sum_{j}^{n}\sum_{k}^{n}bjk(i)$ *j≠k≠i*, and *j* < *k* and n represents the number of shortcuts between node *X* and node *Z*. *bjk(i) = gjk(i)/ gjk*, *gjk* represents the number of shortcuts between the node *k* and node *j*. The ability of a third node *i* to control these two nodes in the exchange of information is represented as *bjk(i)*. It indicates the probability that node *i* is on the shortcut between node *j* and node *k*. Adding together all the absolute betweenness centrality of one node pair that node *i* has in the network results in the absolute betweenness centrality of node *i*, i.e., *c*_*ABi*_. Only with the betweenness centrality *c*_*ABi*_ of nodes that are in the star network is it possible to achieve the maximum value, which is *c*_*Pax*_ in Eq 13.

$$c_{Pax}=(n^{2}-3Q+2)/2$$ (13)

### Data

The actual application process of sentiment analysis of online public opinions cannot rely solely on human resources to complete the selection and screening of massive data on the internet. Therefore, we collected people’s posts regarding online hot events in a forum using web data mining techniques. We selected the "3Q war" event as the study case and collected internet users’ posts under the post whose title is "Collecting signatures to prepare to sue Tencent on behalf of QQ users" on the Tianya forum in 2010 as original data. Tianya forum is an open forum. The times of the posts range from 7:54:50 on 4 November 2010 to 11:29:26 on 13 November 2010. We delete the empty posts and obtain 4,203 reviews for data. The website of the post is http://bbs.tianya.cn/post-free-2015441-1.shtml.

In September 2010, "360 security guards" of QIHU introduced a personal privacy protection tool, "360" privacy protection. This tool claimed QQ software, which belongs to Tencent, spied on users’ privacy. In October 2010, "360" launched the "buttoned bodyguards" security tools to hinder the normal use of QQ software. In November of the same year, Tencent released a "letter to the majority of QQ users" and decided to stop running QQ software on PCs that have "360" software. A commercial war began between the two largest companies, but their users were involved in it. Internet users discussed and condemned their behavior on the major social websites, forming online hot events for that time. This event is called the "3Q war" event.

## Results and Analysis

### Sentiment modes complex network of online public opinions

By calculating the sentiment value of the collected people’s posts, we obtained 4,203 sentiment values, which range from -60 to 60. In the meantime, 94.3 percent of them range from -1 to 1. Then we selected five sentiment symbols as a unit and transformed the sentiment series *C*_*T*_ of online public opinions about "3Q war" event to 4,199 sentiment modes, which consist of 731 types of modes.

**Fig 2:**
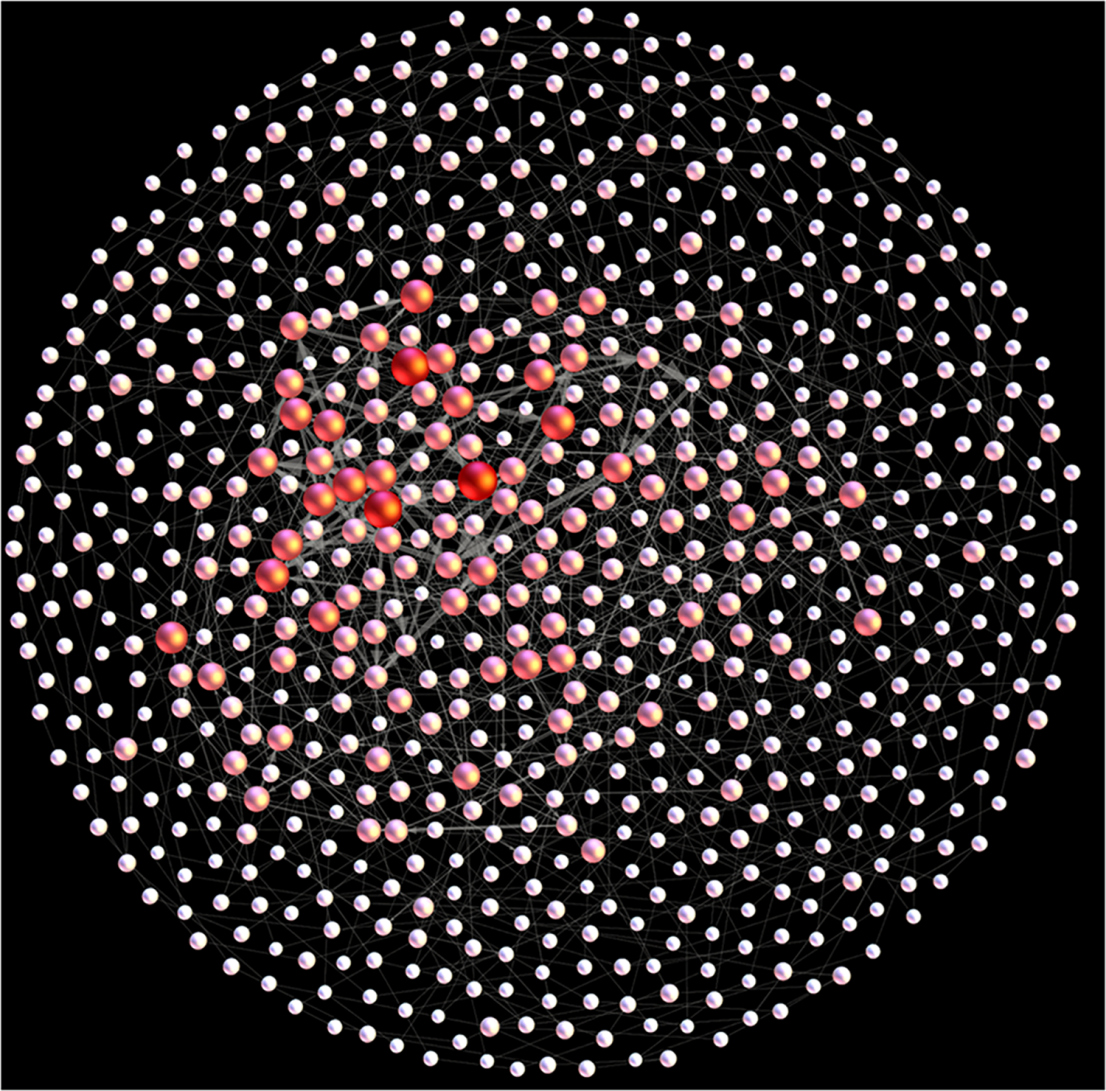
The sentiment mode complex network of online public opinion about "3Q war". The size of a spot represents the strength degree (Eq 7) and the thickness of line represents the size of the weight. The deeper color the node shows, the greater the betweenness centrality (Eq 10) the node has.

To this end, we used a weighted network to describe the connection and the role of each mode in the sentiment series of online public opinions to reflect the change characteristics of people’s sentiments about the "3Q war" event (Fig 2).

### Sentiment modes distribution of online public opinions

**Fig 3:**
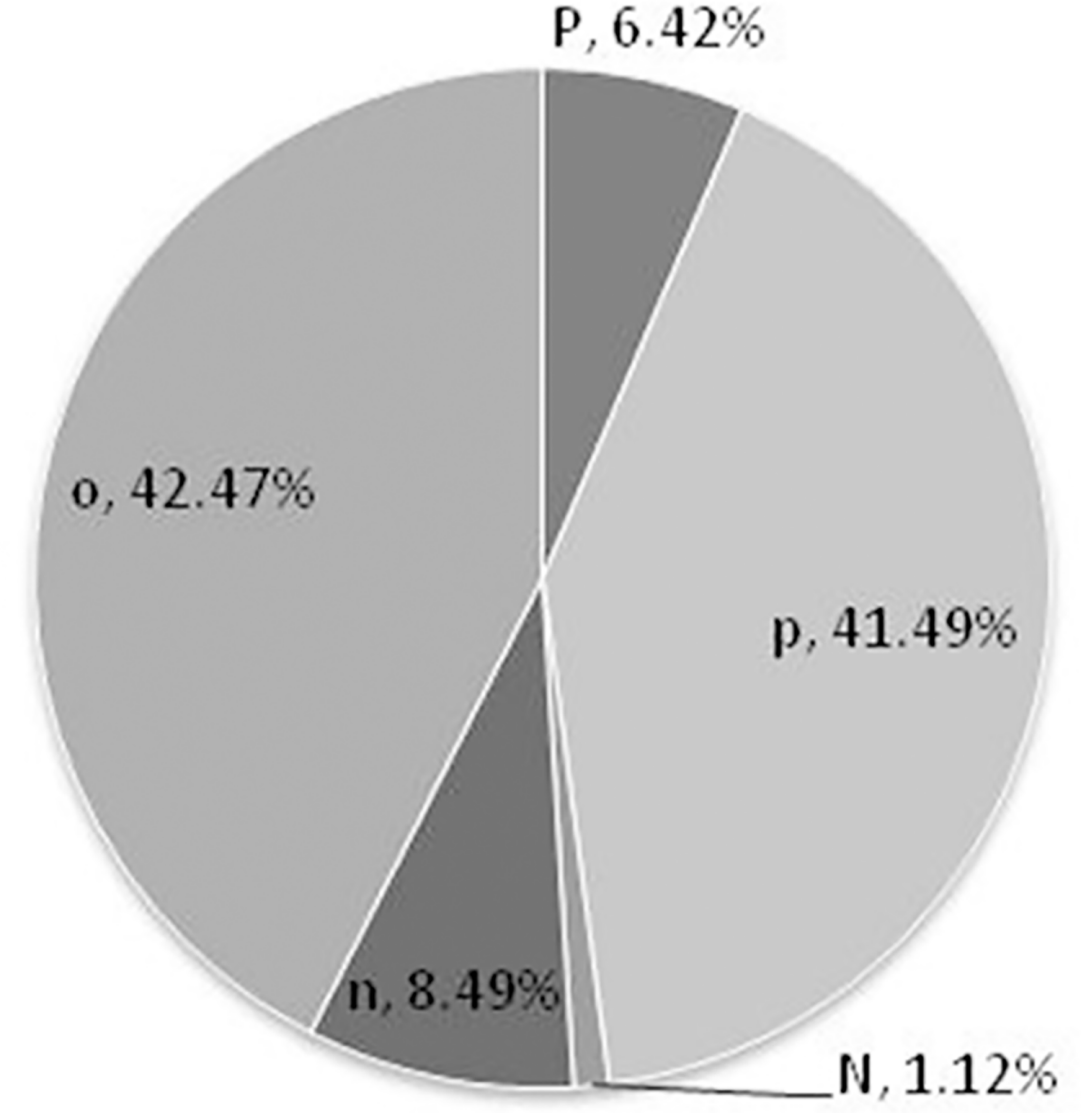
Symbol proportion in the sentiment series of online public opinion about "3Q" war.

We count the sentiment symbols and find that the symbol *p* accounted for 41.5 percent in the entire series. Symbol *p* and symbol *P* accounted for 47.9 percent and symbol *o* accounted for 42.5 percent. The results indicate that the sentiments of the online public about the "3Q" war are mainly positive and neutral. Further, most of those posts are positive. The proportions of sentiment symbols are shown in Fig 3.

We calculated and sorted node strength in the SMCOP. The node strengths of the top 25 nodes, *ooooo*, *ooopo*, *oopop*, *poppp*, *ppopp*, *oppoo*, *ppoop*, *poppo… opopo*, *ppppp*, *ppppo*, *ooppo*, are relatively large. The cumulative node strength distribution reaches 35.4 percent, and the strength distribution of each node is more than 1 percent. The frequency of changes from these 25 mode shifts to other modes, from other mode shifts to these modes and among these modes is relatively high. In addition, through counting the frequency of node strength in the network (Table 4), it can be found that in the 25 larger nodes strength nodes, the characters *P*, *N*, *n* representing strongly positive, strongly negative and weakly negative sentiment respectively does not appear. Weakly positive characters and neutral characters appear symmetrically. The results indicate that the weakly positive and neutral sentiments are the main modes in sentiment mode diffusion of online public opinions and negative modes don’t exist. Therefore, to some extent, it reflects that internet users’ posts tend to convey a positive or neutral sentiment with consistency online hot events. When there are posts with negative sentiments, they will be overwhelmed by posts with positive or neutral sentiments. Internet users who see posts about an event later can be easily guided by a large number of existent sentiments instead of making an objective judgment or even having a critical view.

**Table 4:** The symbol frequency in top 25 nodes of strength degree.

sentiment symbol	frequency
*p*	60
*pp*	9
*ppp*	3
*pppp*	2
*ppppp*	1
*o*	65
*oo*	9
*ooo*	4
*oooo*	2
*ooooo*	1

**Fig 4:**
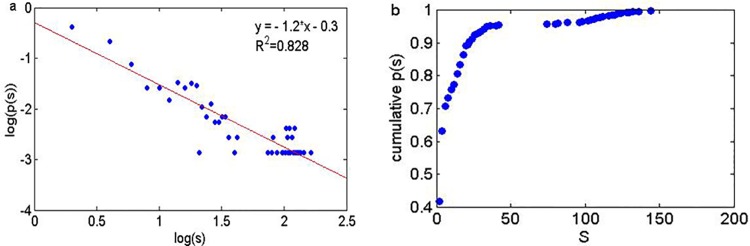
The strength degree distribution in the double logarithmic coordinates and cumulative strength distribution of the SMCOP. (a) The strength distribution in the double logarithmic coordinates of SMCOP. (b) Cumulative strength distribution of SMCOP.

Because of the coarse graining processing, theoretically there are 5^5^ types of modes. However, there are only 731 different types of modes through experiments in the SMCOP. Many nodes in the SMCOP appear repeatedly. To verify if the node strength of the SMCOP obeys a power-law distribution, we analyze the strength distribution of the SMCOP, which is shown in Fig 4. As can be seen from Fig 4, the strength distribution in the double logarithmic coordinates and cumulative strength distribution of SMCOP are more complex. It shows that fewer nodes have higher strength and most nodes have lower strength from a global aspect. We use least square regression to analyze the node strength and cumulative node strength distribution. The regression equation is obtained, i.e., y = -1.2x-0.3, and the goodness of fit is R^2^ = 0.828. The results indicate that the SMCOP tends to obey a power-law distribution as a whole. It also can be seen that the 731 nodes are divided into two parts, i.e., the strength values of the higher first 25 nodes are between 102 and 164. The remaining 704 nodes’ strength values are mainly between 2 and 40.

Through the analysis above, the sentiment modes of online public opinions about the "3Q war" mainly consist of 25 types of continuous neutral and weakly positive sentiment modes and other modes. Both the strength distribution and cumulative node strength distribution of the SMCOP obey a power-law distribution. It indicates that most nodes have smaller strengths, and a few nodes have larger strengths. The online public opinion has same sentiment in the same time, i.e., positive or neutral sentiment.

### Sentiment modes dissemination of online public opinions

**Fig 5:**
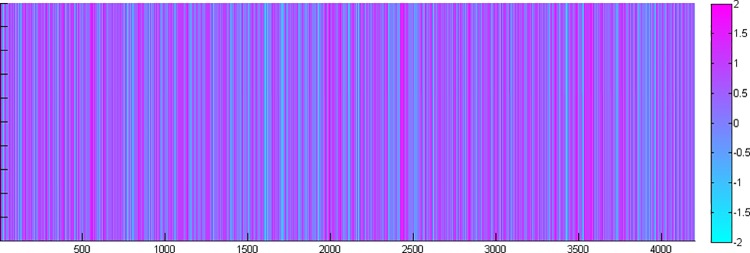
The sentiment evolution color spectrum of online public opinion about "3Q war". The horizontal axis represents the users’ comments in chronological order. The color bar is on the right. Sentiment is divided into positive and negative. A positive sentiment is purple, and a negative sentiment is blue. The deeper the color is, the more intense the sentiment.

The sentiment dissemination of online public opinions about "3Q war" is continuous and alternates with time (Fig 5). To further analyze the sentiment dissemination, we used K-plex method, weighted clustering coefficient and betweenness centrality.

We adopt the K-plex method to find the subgroups in the network. K-plex method does not change the structure of the network significantly because of some nodes’ disappearance in a subgroup. The K-plex method is more focused on the degree of correlation between nodes. When a node scale *g* is 5 and the accommodation coefficient *k* is 2, this method finds ten subgroups in total as shown in Table 5. The sentiment modes in the ten subgroups are primarily neutral and weakly positive rather than negative. These ten mode subgroups can also be divided into two classes. Groups 1 to 9 are the weakly positive class, and group 10 is the neutral class. Deleting the repeated modes in the two classes, the neutral sentiment class has 9 modes, and the weakly positive sentiment class has 5 modes. The modes in the two classes change frequently. In the weakly positive class, other sentiments appear occasionally but turning to weakly positive quickly. In the neutral class, other sentiments also appear occasionally but turning to neutral quickly. These results indicate that sentiments change from single neutral or strongly positive to continuous weakly positive and then turn to single strongly positive in the weakly positive group. They also indicate that sentiments change from single weakly positive, weakly negative or strongly positive to continuous neutral and then turn to single weakly negative, weakly positive or strongly positive in the neutral group.

**Table 5:** Ten mode subgroups found by the K-plex method.

number	Mode subgroups
1	*Poooo ooooo oooon noooo oooop*
2	*Poooo ooooo oooon noooo ooooP*
3	*Poooo ooooo oooon oooop poooo*
4	*Poooo ooooo oooon poooo ooooP*
5	*Poooo ooooo noooo oooop ooooP*
6	*Poooo ooooo oooop poooo ooooP*
7	*ooooo oooon noooo oooop poooo*
8	*ooooo oooon noooo poooo ooooP*
9	*ooooo noooo oooop poooo ooooP*
10	*ppppp ppppo opppp Ppppp ppppP*

To find the core modes in the subgroup, we calculated the weighted clustering coefficient. It considers the weight of the edge while the K-plex method does not. By calculating the weighted clustering coefficient, we can find that only ten modes’ weighted clustering coefficients are not zero, i.e., *ooooo*, *ppppp*, *ooooo*, *ppppP*, *onpon*, *ooooN*, *nponp*, *oooon*, *ooooP*, *Poooo* and *noooo*, which indicates that these ten modes are the core of the network. In addition, based on the weighted clustering coefficient of these ten mode values, the mode *ooooo* is the core of the neutral class and the mode *ppppp* is the core of the weakly positive class. It illustrates that the mutual conversion between modes is not haphazard in the SMCOP but clusters around several types of modes. Further, each class changes dependents on one core mode.

We calculated betweenness centrality to find out the media modes in the network. The modes *ppoop*, *popoP* and *poopp* have the largest betweenness centrality, which illustrates that these three modes play an important role in the topology structure of the SMCOP. Further, conversion between the weakly positive class and neutral class mainly depends on these three modes as intermediaries.

## Conclusions and Future Work

This paper studied the sentiment diffusion of online public opinions about hot events. We adopted the dictionary-based sentiment analysis approach to obtain the sentiment orientation of posts. Based on HowNet and semantic similarity, we calculated each post’s sentiment value and classified those posts into five types of sentiment orientations. Then we used five types of symbols to represent those five types of sentiments. As a result, we obtained a series of sentiment symbol of online public opinions. Meanwhile, the sentiment diffusion is a dynamic complex process. Thus, we applied the complex network to identify the main sentiment status, the main sentiment dissemination pattern and media in the sentiment diffusion process. To make the research feasible, we employed coarse graining method to transfer the abstract sentiment symbol series into sentiment modes so that we constructed a sentiment mode complex network. We selected the online hot event, i.e. "3Q war" event, as the research object and internet users’ posts under the post titled "Collecting signatures to prepare to sue Tencent on behalf of QQ users" on the TianYa forum in 2010 as the study sample.

The sentiment diffusion of online public opinions about "3Q" war event reflects in three aspects. First of all, sentiment modes mainly consist continuous weakly positive and neutral sentiments. The node strength distribution and cumulative node strength distribution of the SMCOP obey power law, in which most nodes have smaller node strengths and a few nodes have larger node strengths. Online public opinion conveys simple positive or neutral sentiment lacking diversity. Secondly, sentiment diffusion process has two classes of subgroups which are weakly positive and neutral classes both with few modes. The modes interchange frequently with the *ppppp* mode and the *ooooo* as intermediary in the weakly positive sentiment subgroup and neutral sentiment subgroup, respectively. The sentiments change from single to continuous and then back to single in the subgroup. Thirdly, there are few high betweenness centrality modes in SMCOP, which have an important intermediary role on the sentiment transmission of online public opinions. When these three modes appear, the sentiment will change thereafter.

Overall, the sentiment of online public opinions regarding hot events such as "3Q war" tends to focus on one or two types. The sentiment converts from a single sentiment into continuous weakly positive or neutral and then back to another single sentiment. The sentiment of internet users’ posts lacks diversity. When the sentiment intermediary appears, we can know that the sentiment will change. It would be helpful for the relevant person or institute to take measures to lead people’s sentiment about online hot events in time by finding the sentiment intermediary.

The "3Q" war event mainly involves the interests of the users of two companies. Therefore, users’ posts mainly focused on their behavior. The sentiments contained in the posts concentrate in two types. The question of whether online hot events in other types have different sentiment distributions and disseminations needs further research.
